# Middle meningeal artery arteriovenous shunting after surgical evacuation of chronic subdural hematoma: a retrospective angiographic cohort study

**DOI:** 10.1007/s10143-026-04411-w

**Published:** 2026-07-21

**Authors:** Malvina Garner, Frederik Fries, Alena Haußmann, Wolfgang Reith, Umut Yilmaz

**Affiliations:** Department of Neuroradiology, Saarland University Hospital, Kirrberger Str, Homburg, Saar 66424 Germany

**Keywords:** Middle meningeal artery, Arteriovenous fistula, Chronic subdural hematoma, Angiography, Craniotomy, Embolization

## Abstract

Arteriovenous shunting involving the middle meningeal artery (MMA) has been reported after cranial trauma, neurosurgical procedures, and endovascular interventions. However, its angiographic frequency and characteristics after chronic subdural hematoma (cSDH) surgery remain poorly defined. This study aimed to assess the frequency and angiographic features of MMA arteriovenous shunting in patients undergoing MMA embolization after surgical evacuation of cSDH. We retrospectively analyzed consecutive patients who underwent selective and superselective MMA angiography during MMA embolization after surgical evacuation of cSDH between January 2020 and March 2023. Angiograms were reviewed for angiographic evidence of arteriovenous shunting, shunt morphology, flow characteristics, venous drainage, relationship to embolization, and angiographic closure after embolization. Surgical technique was classified as burr-hole craniotomy or formal craniotomy. Thirty patients met the inclusion criteria. Angiographic evidence of MMA arteriovenous shunting was identified in 11 patients (36.7%). Shunting was significantly more frequent after craniotomy than after burr-hole craniotomy (90.9% vs. 5.3%, Fisher’s exact test, p < 0.001). Most shunts were detectable before microcatheter advancement, while superselective angiography improved visualization of shunt morphology and venous drainage. All shunts demonstrated low-flow angiographic characteristics without cortical venous reflux. Post-embolization control angiography demonstrated disappearance of the angiographic shunt in all affected patients. Low-flow arteriovenous shunting involving the MMA is a frequent angiographic finding in patients undergoing MMA embolization after cSDH surgery, particularly following craniotomy. These findings provide systematic angiographic characterization of a previously underrecognized postoperative vascular phenomenon. The clinical relevance of such shunting remains uncertain and warrants prospective investigation.

## Introduction

Chronic subdural hematoma (cSDH) is a common neurosurgical condition, particularly affecting elderly patients. Surgical evacuation by burr-hole craniotomy or craniotomy remains a standard treatment, yet postoperative meningeal vascular alterations and their angiographic manifestations remain incompletely characterized [1, 10]. In recent years, middle meningeal artery (MMA) embolization has increasingly been used as an adjunctive or rescue treatment for recurrent or refractory cSDH. Recent evidence, including randomized controlled trials and updated meta-analyses, has further supported MMA embolization as an adjunctive or standalone treatment option in selected patients with cSDH [17]. As a result, selective and superselective MMA angiography is increasingly performed in the clinical management of cSDH, providing an opportunity to detect postoperative dural vascular abnormalities that may previously have remained unrecognized. This development has led to more frequent use of selective and superselective MMA angiography and has therefore increased the opportunity to observe postoperative dural vascular findings.

Recent histological and anatomical studies have substantially advanced the understanding of dural vascular architecture, demonstrating a dense and highly organized arterial and venous network at the micrometer scale. Within this framework, inflammatory and angiogenic mechanisms have been recognized as key drivers of membrane formation and persistence in cSDH [2]. These insights have directly contributed to the rationale for MMA embolization as a treatment strategy for cSDH [11]. Detailed anatomical work has further demonstrated that components of the dural vascular network extend into size ranges that may theoretically be detectable on angiography and has discussed the possibility of physiological arteriovenous connections within the dura mater [13].

Arteriovenous shunting involving the MMA may represent a spectrum of angiographic appearances, ranging from accentuated physiological arteriovenous transit to true low-flow meningeal arteriovenous fistulas. In the present study, such findings were operationally defined by early or focal venous opacification arising from MMA branches, without cortical venous reflux. Supporting the biological plausibility of such a spectrum, histologic evidence for arteriovenous shunts has also been demonstrated in normal dura adjacent to venous sinuses [4].

Meningeal arteriovenous shunts have been described primarily in isolated case reports, most often in association with cranial trauma, skull fractures, neurosurgical procedures, or endovascular interventions [3, 5, 6, 12, 15]. The increasing use of selective and superselective MMA angiography in contemporary cSDH management provides an opportunity to systematically assess the frequency and angiographic characteristics of these findings.

Against this background, the present retrospective angiographic cohort study aims to characterize MMA arteriovenous shunting in the postoperative state following cSDH evacuation. Rather than establishing causality or clinical relevance, the study focuses on systematically describing the frequency, morphology, flow characteristics, detectability, and angiographic behavior of findings consistent with arteriovenous shunting within a post-surgical embolization cohort.

## Materials and methods

### Study design and ethical approval

This retrospective single-center study included patients who underwent MMA embolization following surgical evacuation of cSDH between January 2020 and March 2023. The study was approved by the local German ethics committee (Ethikkommission der Ärztekammer des Saarlandes, Saarbrücken, Germany; No. 221/22). All procedures performed in this study were in accordance with the ethical standards of the 1964 Helsinki Declaration and its later amendments. A retrospective analysis of anonymized clinical and angiographic data was performed.

The manuscript was prepared in accordance with the STROBE recommendations for observational studies. A completed STROBE checklist will be submitted as supplementary material.

### Patient selection

We reviewed all consecutive patients who underwent MMA embolization for cSDH at our institution during the study period. MMA embolization was not performed routinely in all patients undergoing surgery for cSDH during the study period. Rather, patients were referred for MMA embolization after interdisciplinary assessment, typically in the setting of recurrent, residual, or clinically relevant postoperative cSDH, or when an increased risk of recurrence was assumed. The present cohort therefore represents a selected post-surgical embolization cohort and not the overall population of surgically treated cSDH patients.

Inclusion criteria were:

1. Prior surgical evacuation of cSDH by burr-hole craniotomy or craniotomy;
2. Availability of selective MMA angiography;
3. Availability of superselective MMA angiography before embolization.

Exclusion criteria were:

1. MMA embolization without prior surgical evacuation of cSDH;
2. Absence of superselective MMA angiography.

From an initial cohort of 35 consecutive patients undergoing MMA embolization during the study period, five patients were excluded from analysis: three due to absence of prior surgical evacuation of cSDH and two due to lack of available superselective MMA angiography. The final study cohort therefore comprised 30 patients. Because the study was designed as an angiographic analysis, only clinical variables that were consistently available in the procedural and imaging records were included. Detailed information on trauma history, interval between trauma and hematoma development, anticoagulant or antiplatelet medication, arterial hypertension, comorbidities, and prior cranial procedures was not systematically available for all patients and was therefore not included in the statistical analysis.

The total number of patients who underwent surgical evacuation for cSDH during the study period could not be reliably determined from the institutional database. Therefore, the present study reports the frequency of MMA arteriovenous shunting within the post-surgical MMA embolization cohort, not within the overall surgically treated cSDH population.

### Surgical technique classification

Surgical technique was classified as burr-hole craniotomy or formal craniotomy. Burr-hole craniotomy was defined as a trephination diameter ≤ 30 mm, whereas formal craniotomy was defined as a bone opening > 30 mm, consistent with commonly used neurosurgical definitions [14, 16]. Subdural drainage was placed in all cases.

### Angiographic and embolization procedures

All angiographic procedures were performed using a Siemens Axiom Artis or Siemens Artis Q digital subtraction angiography system (Siemens Healthcare, Erlangen, Germany). Selective catheterization of the external carotid artery and MMA was followed by superselective catheterization using DMSO-compatible microcatheters. Catheter position varied according to vascular anatomy and procedural requirements and was determined at the discretion of the treating interventionalist. Catheter positions ranged from proximal MMA segments to more distal MMA branches.

All contrast injections were performed manually. Power injections were not used.

Angiography and MMA embolization were performed under general anesthesia.

MMA embolization was performed using Onyx (Medtronic, Irvine, CA), with the goal of occluding MMA branches supplying the cSDH membranes according to the treating interventionalist’s assessment.

### Angiographic assessment

Selective and superselective MMA angiograms were reviewed for angiographic findings consistent with arteriovenous shunting. For the purpose of this study, arteriovenous shunting was defined as early or focal venous opacification arising from MMA branches, with or without a tram-track configuration of parallel arterial and venous channels.

For each patient, the following angiographic variables were systematically recorded:

1. Presence or absence of arteriovenous shunting;
2. Detectability on selective angiography;
3. Detectability on superselective angiography;
4. Anatomical location of the shunt;
5. Shunt morphology, including tram-track configuration or focal venous pouch;
6. Venous drainage pattern;
7. Presence or absence of cortical venous reflux;
8. Qualitative flow characteristics;
9. Procedure-related or non–procedure-related appearance;
10. Angiographic occlusion after embolization.

Shunts were classified as procedure-related if they were absent on initial selective or superselective angiographic runs but appeared during the embolization procedure. Shunts were classified as non–procedure-related if they were visible on selective external carotid, selective MMA, or initial superselective MMA angiography before microcatheter advancement into the segment from which the shunt originated or before embolic injection.

All angiographic assessments were performed in consensus by two neuroradiologists. All shunts were qualitatively classified as low-flow or high-flow. Features suggesting aggressive venous drainage, including rapid cortical venous reflux, were specifically assessed.

### Timing of angiography

The interval between surgical evacuation of cSDH and angiographic evaluation was recorded for all patients.

### Data collection

Patient demographics, available surgical details, and angiographic findings were extracted from medical records, procedural reports, and angiographic imaging data. Clinical outcomes, recurrence rates, and long-term follow-up were not systematically available and were therefore not analyzed.

### Statistical analysis

Statistical analysis was performed using SPSS version 25.0 (IBM Corp., Armonk, NY). Categorical variables were compared using Fisher’s exact test because of the small sample size and low expected cell counts. Continuous variables were reported descriptively. A p-value < 0.05 was considered statistically significant. All statistical analyses were exploratory and descriptive in nature, and the study was not powered for inferential conclusions.

## Results

### Patient characteristics and surgical techniques

The final study cohort comprised 30 patients, including 24 men and 6 women. The mean age was 75.3 ± 2.1 years.

Burr-hole craniotomy was performed in 19 patients, and formal craniotomy was performed in 11 patients. In the burr-hole group, the mean trephination diameter was 27.7 ± 2.2 mm. In the craniotomy group, the mean bone opening diameter was 47.4 ± 23.3 mm. Subdural drainage was placed in all cases.

### Frequency of arteriovenous shunting

Angiographic evidence of MMA arteriovenous shunting was identified in 11 of 30 patients (36.7%). Shunting occurred in 1 of 19 patients (5.3%) following burr-hole craniotomy and in 10 of 11 patients (90.9%) following formal craniotomy. This difference was statistically significant (Fisher’s exact test, *p* < 0.001).

### Angiographic characteristics

All identified shunts demonstrated low-flow angiographic characteristics. These included subtle early anterograde venous drainage into meningeal veins, absence of markedly dilated arterial feeders, and absence of cortical venous reflux. No high-flow shunt was observed.

Most shunts were located in distal MMA branches. In several cases, the shunt was located near the presumed surgical site, as inferred from the relationship to burr-hole or craniotomy margins and the corresponding MMA territory. A tram-track configuration, defined as parallel visualization of arterial and venous channels along the MMA territory, was observed in representative cases. Focal venous pouches or spot-like venous opacification were also identified in selected cases.

**Fig. 1:**
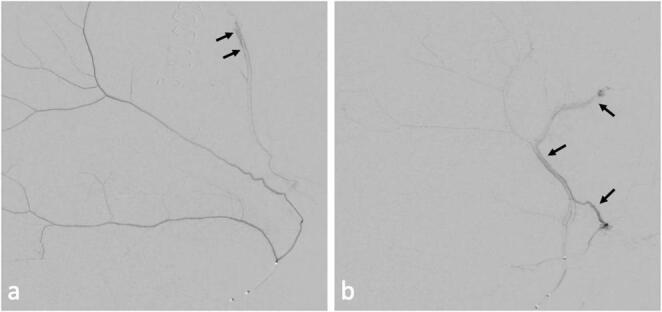
Examples of arteriovenous shunting on superselective MMA angiography after surgical evacuation of chronic subdural hematoma. Arrows indicate early venous opacification and tram-track configuration. **a** Superselective angiogram of the MMA demonstrating low-flow arteriovenous shunting with a characteristic tram-track configuration, reflecting parallel visualization of arterial and venous channels. Early anterograde venous drainage is seen adjacent to distal MMA branches. **b** Superselective MMA angiogram from a different patient showing a similar angiographic pattern of low-flow arteriovenous shunting. The tram-track configuration is again evident, with subtle early venous opacification along the MMA territory, consistent with meningeal arteriovenous shunting

Representative examples of low-flow arteriovenous shunting of the MMA after surgical evacuation of cSDH on superselective angiography are shown in Fig. 1.

### Procedure-related and non–procedure-related shunting

Two arteriovenous shunts were identified as appearing de novo during embolization procedures. In these cases, the angiographic shunt originated from the MMA segment corresponding to the microcatheter position and was not visible on preceding angiographic runs. These findings were classified as procedure-related angiographic shunts.

The remaining nine shunts were considered non–procedure-related because angiographic evidence of shunting was already present before embolization and before microcatheter manipulation of the corresponding distal MMA segment. These shunts were typically located in distal MMA branches.

### Detectability on selective and superselective angiography

Arteriovenous shunting was appreciable on selective angiography in the majority of non–procedure-related cases. However, in 3 of 9 non–procedure-related shunts (33.3%), shunting was not detectable on selective angiography due to subtle low-flow characteristics and became evident only on superselective MMA injections.

**Fig. 2:**
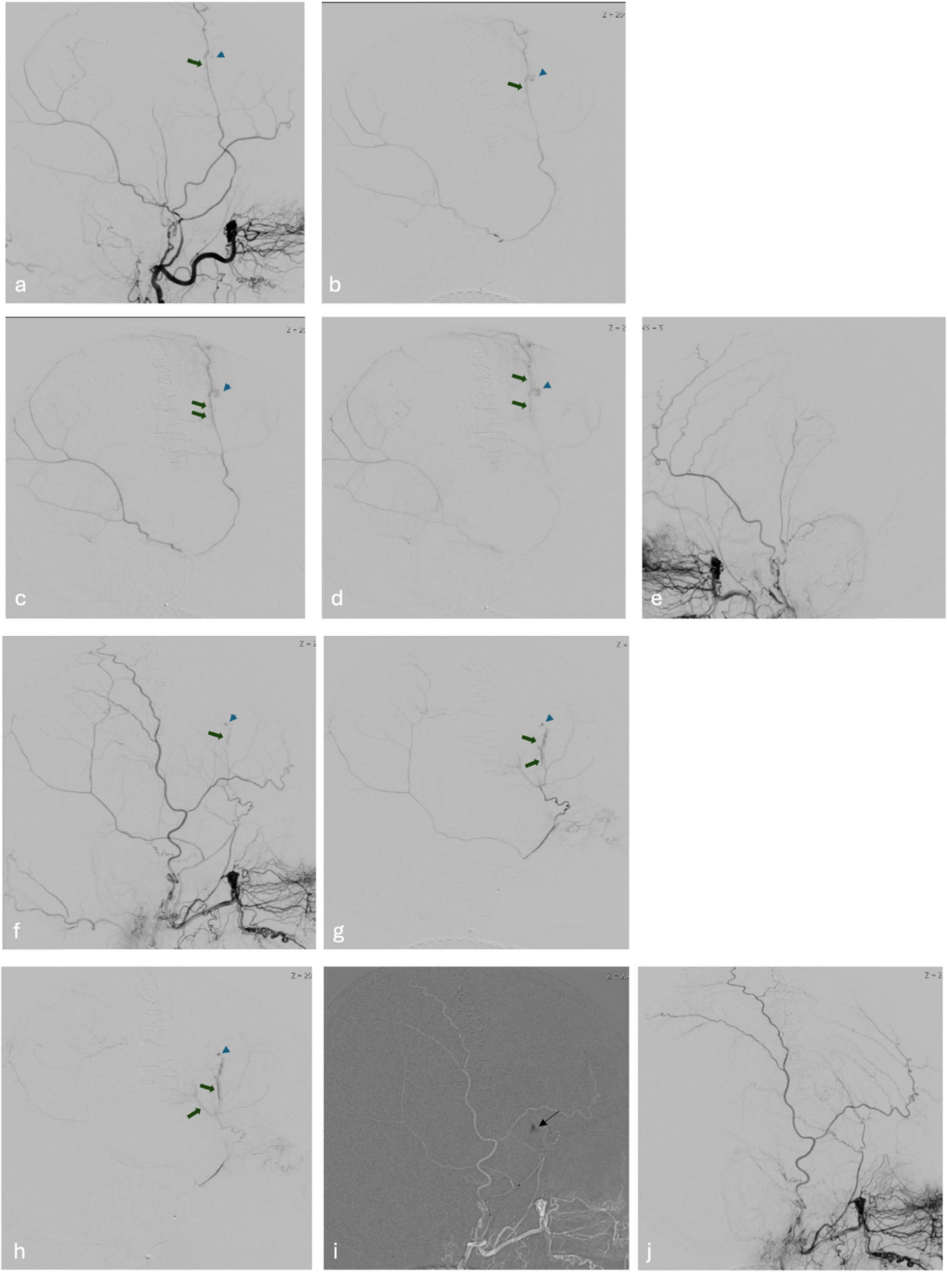
Bilateral MMA arteriovenous shunting in a single patient. Selective and superselective angiographic findings in a patient with bilateral low-flow arteriovenous shunting involving the frontal branches of the MMA following surgical evacuation of chronic subdural hematoma. Arrows and arrowheads highlight the key angiographic findings, including focal venous pouch, early venous opacification, tram-track configuration, and disappearance of the shunt after embolization. Left side: **a** Selective angiography of the left MMA with the catheter positioned in the maxillary artery demonstrates early venous drainage arising from the distal frontal branch of the MMA. A small nodular venous pouch and a subtle tram-track configuration are appreciable, consistent with arteriovenous shunting. **b**–**d** Superselective angiography of the left MMA with the microcatheter positioned in the proximal MMA segment allows improved visualization of shunt morphology and hemodynamics. Early arterial, arterial, and late arterial phases show progressive opacification of the arteriovenous shunt with early venous drainage along the MMA territory. **e** Post-embolization angiography following embolization with liquid embolic material demonstrates proximal occlusion of the MMA and angiographic closure of the arteriovenous shunt. Right side: **f** Selective angiography of the right MMA reveals a subtle focal contrast accumulation in the distal frontal branch without unequivocal early venous drainage. **g**–**h** Superselective angiography with the microcatheter positioned in the proximal MMA segment demonstrates early venous opacification corresponding to the previously subtle focal contrast accumulation. A tram-track configuration becomes increasingly conspicuous from the early arterial phase through the arterial and late arterial phases, delineating venous drainage along the MMA territory. **i** Arterial roadmap imaging demonstrates early venous blush consistent with arteriovenous shunting. **j** Post-embolization angiography confirms complete occlusion of the right MMA and angiographic closure of the arteriovenous shunt

Superselective angiography consistently provided superior visualization of shunt morphology and venous drainage compared with selective injections alone. In particular, tram-track configurations and subtle early venous drainage were more clearly delineated on superselective angiographic runs. An example illustrating improved shunt visualization by superselective angiography is shown in Fig. 2. 

### Angiographic result after embolization

Following MMA embolization, post-embolization control angiography demonstrated disappearance of the angiographic shunt in all patients with identified arteriovenous shunting. No residual shunting was observed on final control angiography.

### Timing of angiography

The mean interval between surgical evacuation of cSDH and angiographic evaluation was 6.5 ± 3.4 days in patients with arteriovenous shunting and 5.1 ± 3.1 days in patients without shunting. Because of the exploratory nature of the study and the limited sample size, this continuous variable was reported descriptively.

## Discussion

### Principal findings

In this retrospective angiographic cohort study of patients undergoing MMA embolization after surgical evacuation of cSDH, angiographic evidence of arteriovenous shunting involving the MMA was identified in more than one third of cases. These findings were markedly more frequent following craniotomy compared with burr-hole craniotomy. All shunts demonstrated low-flow angiographic characteristics without cortical venous reflux, and post-embolization control angiography demonstrated disappearance of shunting in all affected patients.

Importantly, arteriovenous shunting was detectable before microcatheter manipulation of the corresponding MMA segment in the majority of cases, indicating that most shunts were not procedure-related. Superselective angiography improved detectability and characterization of shunt morphology compared with selective angiography alone.

### Relation to prior literature

True meningeal arteriovenous fistulas of the MMA have historically been regarded as rare entities, described predominantly in isolated case reports or small series, most often in association with cranial trauma, skull fractures, or neurosurgical procedures [3, 5]. MMA arteriovenous shunting has also been described in the context of cSDH and its treatment, including cases in which meningeal arteriovenous shunting was detected during or after MMA embolization [12, 15], and cases of cSDH associated with an MMA arteriovenous fistula treated by embolization plus burr-hole drainage [6]. Across these reports, shunts were typically low-flow, lacked cortical venous reflux, and were often detected incidentally.

In contrast to the perceived rarity of meningeal arteriovenous shunting in earlier literature, the proportion observed in the present cohort was substantially higher. This discrepancy should be interpreted cautiously and does not necessarily imply a true increase in incidence. A plausible explanation is a change in case ascertainment: contemporary cSDH management increasingly involves MMA embolization, which routinely includes selective and superselective MMA angiography. This practice may uncover subtle low-flow shunting that would otherwise remain inapparent. Consistent with this interpretation, one third of non–procedure-related shunts in our cohort were not appreciable on selective angiography and became evident only after superselective catheterization.

Critically, this remains a hypothesis. Earlier reports do not provide uniform angiographic protocols and therefore do not allow definitive conclusions regarding how often superselective MMA injections were systematically performed or how often subtle low-flow shunts were actively sought.

### Angiographic characterization and low-flow nature

All shunts identified in this study demonstrated angiographic features consistent with low-flow meningeal arteriovenous shunting, including subtle early anterograde venous drainage into meningeal veins, absence of markedly dilated arterial feeders, and lack of cortical venous reflux. No angiographic features of aggressive high-flow dural arteriovenous fistulas were observed.

This angiographic phenotype is in line with the low-aggressiveness spectrum described in historical MMA fistula series, where venous drainage was often confined to the meningeal venous system rather than cortical veins [3, 5]. In this context, angiographic signs frequently discussed in the literature, such as tram-track configuration of parallel arterial and venous channels and focal venous pouches, were also observed in our cohort. These findings support the interpretation that the detected lesions represent low-flow meningeal arteriovenous shunting rather than aggressive dural arteriovenous fistulas.

Importantly, the angiographic appearance of low-flow meningeal shunting must be interpreted within a broader spectrum of meningeal vascular alterations. Miki et al. [9] reported an association between meningeal arteriovenous shunting, increased membrane vascularity, and recurrence in cSDH, suggesting that such shunting may reflect an underlying hypervascular and angiogenically active dural environment. At the extreme end of this spectrum, rare occult vascular malformations such as micro-AVMs have been described as a cause of subdural hemorrhage and may only become detectable on superselective angiography [8].

In this context, our observation that one third of non–procedure-related shunts were not appreciable on selective angiography but became evident only on superselective MMA injections underscores the role of superselective angiography in detecting subtle low-flow meningeal vascular alterations.

#### Anatomical and physiological considerations

Recent anatomical and histological work has revised the traditional view of the dura mater as a relatively avascular structure. Shapiro and colleagues emphasized the complex cranial dural vasculature and discussed the possibility of physiological arteriovenous connections within the outer dural vascular network [13]. Histologic evidence for arteriovenous shunts in normal dura has also been reported [4]. Taken together, these data support the concept that angiographic MMA arteriovenous shunting may represent a spectrum ranging from accentuated physiological arteriovenous transit to true low-flow meningeal fistulas, potentially modulated by postoperative inflammation and angiogenesis in the cSDH setting [2].

The two procedure-related shunts observed in this study may also be interpreted within this spectrum. Rather than necessarily representing newly created fistulas, they could reflect the opening or unmasking of pre-existing small dural arteriovenous anastomoses during selective contrast injection, changes in local pressure gradients, or embolization-related flow redistribution.

### Association with surgical technique

A notable finding of this study is the association between arteriovenous shunting and formal craniotomy compared with burr-hole craniotomy. However, this finding must be interpreted cautiously. Burr-hole craniotomy is typically used for uncomplicated, liquefied cSDH, whereas formal craniotomy is generally reserved for more complex, organized, or septated hematomas. Therefore, the observed association may not only reflect the size of the bone opening or the degree of dural manipulation, but also differences in hematoma complexity, organization, inflammatory activity, or case selection. While causality cannot be inferred, larger bone openings and more extensive dural manipulation during formal craniotomy may plausibly be associated with detectable dural vascular alterations or accentuation of pre-existing arteriovenous connections.However, alternative explanations must be considered. cSDH is frequently associated with antecedent minor trauma, and trauma is an established mechanism for MMA fistula formation [3, 5]. Given the retrospective design and the absence of preoperative angiography, the relative contributions of trauma, surgery, postoperative vascular remodeling, and endovascular manipulation cannot be disentangled.

#### Procedure-related shunting

Two shunts were identified as appearing de novo during embolization procedures and were classified as procedure-related. Even in these cases, angiographic features remained consistent with low-flow meningeal shunting without cortical venous reflux or hemorrhagic blush. Similar observations have been reported in isolated cases, where changes in flow dynamics, pressure gradients, or endothelial integrity during embolization were proposed as potential mechanisms [12, 15]. The rarity of such events in our cohort suggests that procedure-related shunting represents a minority of cases.

#### Timing of angiography

The interval between surgical evacuation and angiographic evaluation did not differ significantly between patients with and without arteriovenous shunting. This finding argues against a strong temporal dependency of shunt detectability within the early postoperative period and suggests that timing alone does not explain the presence or absence of angiographic shunting in this cohort.

#### Clinical implications

The clinical significance of low-flow MMA arteriovenous shunting remains uncertain. In our cohort, all shunts disappeared on final post-embolization control angiography during the same intervention; however, no conclusions can be drawn regarding symptoms, hemorrhagic risk, recurrence, durability of occlusion, or necessity of treatment, given the absence of systematic clinical outcome data and follow-up angiography. Consistent with prior reports, low-flow meningeal arteriovenous shunts have generally been regarded as benign angiographic findings, and spontaneous closure has been described in selected cases [7], although rare hemorrhagic complications have also been reported.

From a practical standpoint, our findings suggest that superselective MMA angiography substantially improves the detection and characterization of meningeal arteriovenous shunting and may be considered when detailed evaluation of MMA angioarchitecture is clinically relevant. At the same time, these findings should not be overinterpreted as aggressive vascular lesions in the absence of high-flow characteristics or cortical venous reflux.

### Limitations

This study has several important limitations. Its retrospective, single-center design and modest sample size limit generalizability. The lack of preoperative angiography precludes determination of whether shunts were pre-existing, trauma-related, surgery-induced, related to postoperative vascular remodeling, or procedure-related. The interval between initial trauma and the development of cSDH was not systematically available, although trauma is a known potential mechanism of MMA arteriovenous shunt formation. In addition, detailed clinical variables such as anticoagulant or antiplatelet medication, arterial hypertension, comorbidities, hematoma organization, and prior cranial procedures were not consistently available and could not be included in the analysis. The study population represents a selected cohort of patients undergoing MMA embolization after cSDH surgery, introducing inherent selection bias. Therefore, the reported frequency should not be interpreted as the prevalence of MMA arteriovenous shunting in all surgically treated cSDH patients.

In addition, the total number of surgically treated cSDH patients during the study period could not be reliably determined. Clinical outcomes, recurrence rates and long-term follow-up were not systematically available, preventing assessment of clinical relevance. No follow-up angiography was available to assess the durability of shunt occlusion beyond the final angiographic run of the embolization procedure. Although angiograms were reviewed according to predefined criteria, differentiating true low-flow arteriovenous fistulas from accentuated early venous opacification or other subtle angiographic flow phenomena may be challenging. Finally, statistical analyses were exploratory and should not be interpreted as confirmatory.

## Conclusion

Angiographic evidence of low-flow arteriovenous shunting involving the MMA is a frequent finding in patients undergoing MMA embolization after surgical evacuation of cSDH, particularly following craniotomy. These shunts predominantly exhibit benign low-flow characteristics without cortical venous reflux and are often best visualized on superselective angiography. While their pathophysiology and clinical significance remain uncertain, the present study provides a systematic angiographic characterization and frequency estimate that may serve as a foundation for future prospective investigations. 

## Data Availability

The datasets generated and analyzed during the current study are not publicly available due to institutional data protection regulations and patient privacy considerations, as they contain clinical and angiographic patient data. De-identified data may be made available from the corresponding author upon reasonable request and after approval by the responsible institutional authorities and ethics committee, where applicable.
